# Oscillatory Bursting as a Mechanism for Temporal Coupling and Information Coding

**DOI:** 10.3389/fncom.2020.00082

**Published:** 2020-09-15

**Authors:** Idan Tal, Samuel Neymotin, Stephan Bickel, Peter Lakatos, Charles E. Schroeder

**Affiliations:** ^1^Department of Psychiatry, Columbia University Medical Center, New York, NY, United States; ^2^Translational Neuroscience Division, Center for Biomedical Imaging and Neuromodulation, Nathan Kline Institute for Psychiatric Research, New York, NY, United States; ^3^Feinstein Institutes for Medical Research, Northwell Health, New York, NY, United States; ^4^Departments of Neurosurgery and Neurology, Northwell Health, New York, NY, United States; ^5^Department of Psychiatry, New York University School of Medicine, New York, NY, United States

**Keywords:** oscillations, transients, bursts, timing, single trial, methods

## Abstract

Even the simplest cognitive processes involve interactions between cortical regions. To study these processes, we usually rely on averaging across several repetitions of a task or across long segments of data to reach a statistically valid conclusion. Neuronal oscillations reflect synchronized excitability fluctuations in ensembles of neurons and can be observed in electrophysiological recordings in the presence or absence of an external stimulus. Oscillatory brain activity has been viewed as sustained increase in power at specific frequency bands. However, this perspective has been challenged in recent years by the notion that oscillations may occur as transient burst-like events that occur in individual trials and may only appear as sustained activity when multiple trials are averaged together. In this review, we examine the idea that oscillatory activity can manifest as a transient burst as well as a sustained increase in power. We discuss the technical challenges involved in the detection and characterization of transient events at the single trial level, the mechanisms that might generate them and the features that can be extracted from these events to study single-trial dynamics of neuronal ensemble activity.

## Introduction

At a most basic level, neuronal oscillations reflect synchronous and rhythmic shifting of neuronal ensembles between high and low excitability states (Buzsaki, 2006; Schroeder and Lakatos, 2009). An obvious consequence is that most neurons in an ensemble are more likely to fire action potentials at a particular (high excitability) oscillatory phase. Neuronal oscillations have been proposed to underlie many critical brain operations including attentional selection of sensory input (Schroeder and Lakatos, 2009), parsing/chunking of complex input streams (Poeppel et al., 2008; Ding and Simon, 2014), generation of motor output (Baker et al., 1999; Parkkonen et al., 2015), memory encoding and retrieval (Jensen et al., 2007), ordering of information carried by spike trains through spike-phase coding (Kayser et al., 2009) and temporal coupling of distant ensembles to enhance information transfer (Varela et al., 2001; Fries, 2015; Singer, 2018). Key to their mechanistic role in these operations is the idea that neuronal oscillations in a particular frequency synchronize dynamically to couple a group of neurons into a cell assembly for a specific brain task (Buzsáki, 2010), and then just as dynamically desynchronize so that neurons can regroup for the next brain task.

While oscillatory activity is often viewed as sustained, this perspective has been challenged in recent years by the notion that oscillations may occur as transient bursts of activity that may only appear as sustained activity when averaging across multiple trials (Lakatos et al., 2004; Jones, 2016; Sherman et al., 2016), in contrast to the idea that averaging in the time domain can diminish or obliterate oscillations when they are not phase-aligned across trials. Since some brain tasks last a few milliseconds (e.g., reacting to an alerting stimulus), while others require many seconds or even longer (solving a mathematical equation), both scenarios are likely to co-exist.

In this review, we will first contrast the ideas of oscillations as transient bursts vs. sustained events, and outline the technical challenges involved in the detection and characterization of transient events at the single trial level. After that we will discuss the circumstances and mechanisms that likely determine whether an oscillation will emerge as a transient or a more sustained brain event.

## Oscillatory Events

### Methods to Identify Transient Oscillatory Bursts

**Figure 1:**
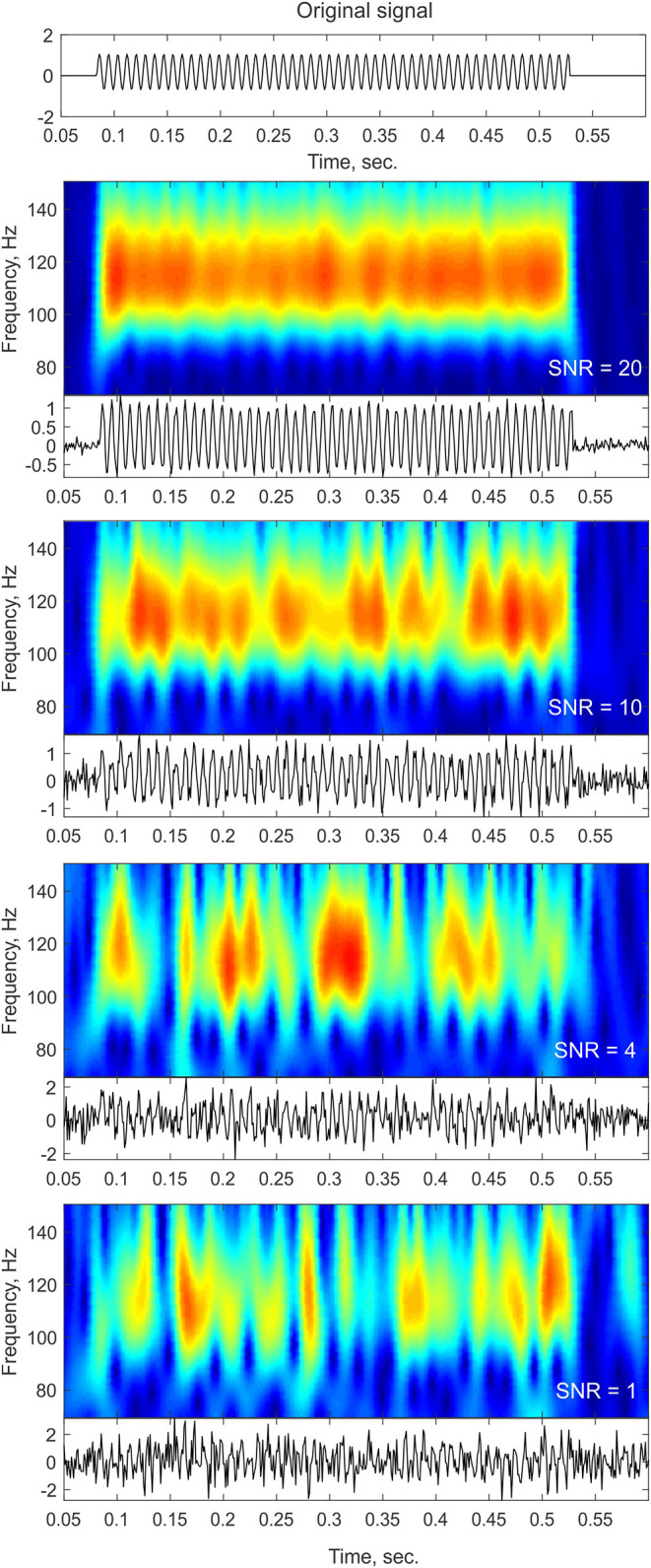
Noise influence on time-frequency profiles. The top panel shows a trace of a pure sinusoid at 110 Hz with a constant amplitude lasting for a duration of ~400 ms. The bottom panels show the time-frequency representation of the signal (top insets) and the time-domain trace (bottom insets) after adding different levels of white Gaussian noise. As the noise level increases (lower SNR), the time-frequency profile contains more gaps and becomes more “bursty” in appearance. In cases of low SNR, such as the ones that might be observed in single trial or ongoing data, sustained oscillations might be broken down into isolated peaks and thus might be considered as transient bursts that might even slightly vary in frequency as a result of the noise structure.

One of the main challenges in single trial analysis is dealing with low signal-to-noise ratio (SNR). The idea behind averaging across trials is that the signals related to some event are enhanced compared to neural activity unrelated to the event, and other non-neural sources of noise, thus providing a representative signal for a “clean” neural response to the stimulus. Obviously, however, the brain has to operate on a single trial basis when performing a cognitive task. If indeed transient oscillatory bursts are involved in information processing, the first step would be to reliably detect such transients at the single trial level. However, as evident from the methods described below, reliable detection of power increases at the single trial level is not trivial. Due to the typically low SNR of single trial responses, frequency decomposition can yield a “bursty” time-frequency profile even in the simple case of constant-amplitude sustained oscillations (see Figure 1). This effect is particularly likely to occur when there is cross-frequency phase amplitude coupling (Lakatos et al., 2005; Schroeder and Lakatos, 2009) and an oscillation is obscured by noise in the non-ideal phase of the lower-frequency oscillation.

Due to the technical challenge presented by the low single-trial SNR, several methods for transient detection have been proposed. Most detection algorithms rely on filtering the data into frequency bands (with a wavelet convolution or a Hilbert transform) and detecting whether a power fluctuation exceeds an amplitude threshold on a trial-by-trial basis. This is known as the p-episode method and may also be combined with a duration threshold (Caplan et al., 2001). Variations of this method have been used to study single trial oscillations in recent years. Sherman et al. (2016) detected beta transients by finding the maxima in the single trial wavelet transformed data. The authors chose the highest beta event in each trial, sorted the events from low to high power and analyzed the top 50 highest power events. This procedure revealed a stereotypical time domain waveform that spans <150 ms of a beta burst (roughly three cycles). Lundqvist et al. (2016) used a similar approach to detect an increase in gamma power of two standard deviations above the mean spectral power in that band, but also added a duration constraint of an increase lasting at least three cycles. Neymotin et al. (2020a) demonstrates that below three cycles, any “length measure” becomes unreliable, in that it overestimates the number of cycles. Hughes et al. (2012) used an oscillation detection method to extract both sustained and transient rhythms from rat hippocampal recordings termed Better Oscillation Detection Method [BOSC; first described by Caplan et al. (2001)]. The BOSC method is applied to continuous signals to detect the incidence of oscillatory components that exceed amplitude and duration thresholds while ignoring the transient voltage fluctuations that may accompany artifacts or evoked potentials. The power threshold is set as the 95th percentile of the theoretical χ^2^ distribution of wavelet power values and the duration threshold was set to three cycles (again, similar to previous methods). An extension to the BOSC method was recently suggested (eBOSC; Kosciessa et al., 2019) in which rhythmic and non-rhythmic episodes are automatically separated. An additional measure of “rhythmicity” termed lagged-coherence uses the present phase of a signal to predict future phases (Fransen et al., 2015). They show rhythmicity peaks detected in ongoing sensorimotor signals that are not visible using conventional power analysis, suggesting that rhythmicity measures are more suitable for identifying neuronal oscillations. Another approach to the detection and characterization of neuronal rhythms uses Hidden Markov Models (HMMs) to overcome some of the limitations of the amplitude-threshold approaches by avoiding a direct amplitude envelope threshold (Quinn et al., 2019). The HMM represents the signals as a system that moves through a set of discrete states, with each state having a probability of being “on” at each time point. Thus, the thresholding procedure is applied to the probabilities rather than the signals themselves. In addition, using temporal regularization, HMM can avoid state transitions due to small changes in the envelope close to the threshold [see Figure 2 in Quinn et al. (2019)]. One of the downsides of this method is that a fixed number of states must be defined in advance. In cases where the distribution of power values (or probabilities) is bi-modal, it is easy to define two states, but in many cases it is harder to define and interpret several states, specifically when studying wide-band phenomenon.

Single trial analysis and power-change detection can be computationally costly and might not be feasible for real-time closed-loop brain stimulation experiments or brain-machine interfaces. Karvat et al. (2019) suggested a method for the detection of transient oscillatory activity specifically designed for real-time data analysis and demonstrated its usefulness for analyzing volitional increase of beta-band burst-rate in the motor cortex of rats. The authors suggest defining a burst as a power peak in time and frequency, exceeding a threshold defined as a percentile to assure a statistically sound significance definition under non-normal distributions. The method is based on 32 real-time narrow bandpass FIR filters followed by peak and trough detection in the filtered signals that exceed the threshold set as the 98th percentile of power.

In addition to the spectrotemporal properties of oscillations, waveform shapes appear to matter as well. Robust differences in the waveform shapes of the oscillations mentioned above can be assumed to represent differences in the properties of their underlying generators (for review see Cole and Voytek, 2017). Due to the rich and possibly variant waveform across different cortical locations and cognitive tasks, detection techniques should combine power threshold, duration threshold and waveform specificity for each frequency band and recording location. An example of such an approach is to detect increases in the single trial time-domain or time-frequency data, then calculate the principal components of the time-domain waveforms and use the first principal component as a template for the detection of additional events in the time domain using a template matching scheme (see Abeles, 2014; Tal and Abeles, 2016, 2018). In brief, a segment of data is projected onto the template. The length of the projection is treated as the signal and the residual is treated as the noise. The threshold is then based on the signal-to-noise ratio. Such methods may lead to the creation of a “dictionary” of waveforms (similar to EEG atlases; Stern, 2005) that exhibit different oscillatory properties. Generating such a dictionary of oscillatory signatures may allow us to further test interactions between different neuronal populations under the assumption that a specific signature is generated by a specific cell population or a specific process (Siegel et al., 2012; Womelsdorf et al., 2014).

**Figure 2:**
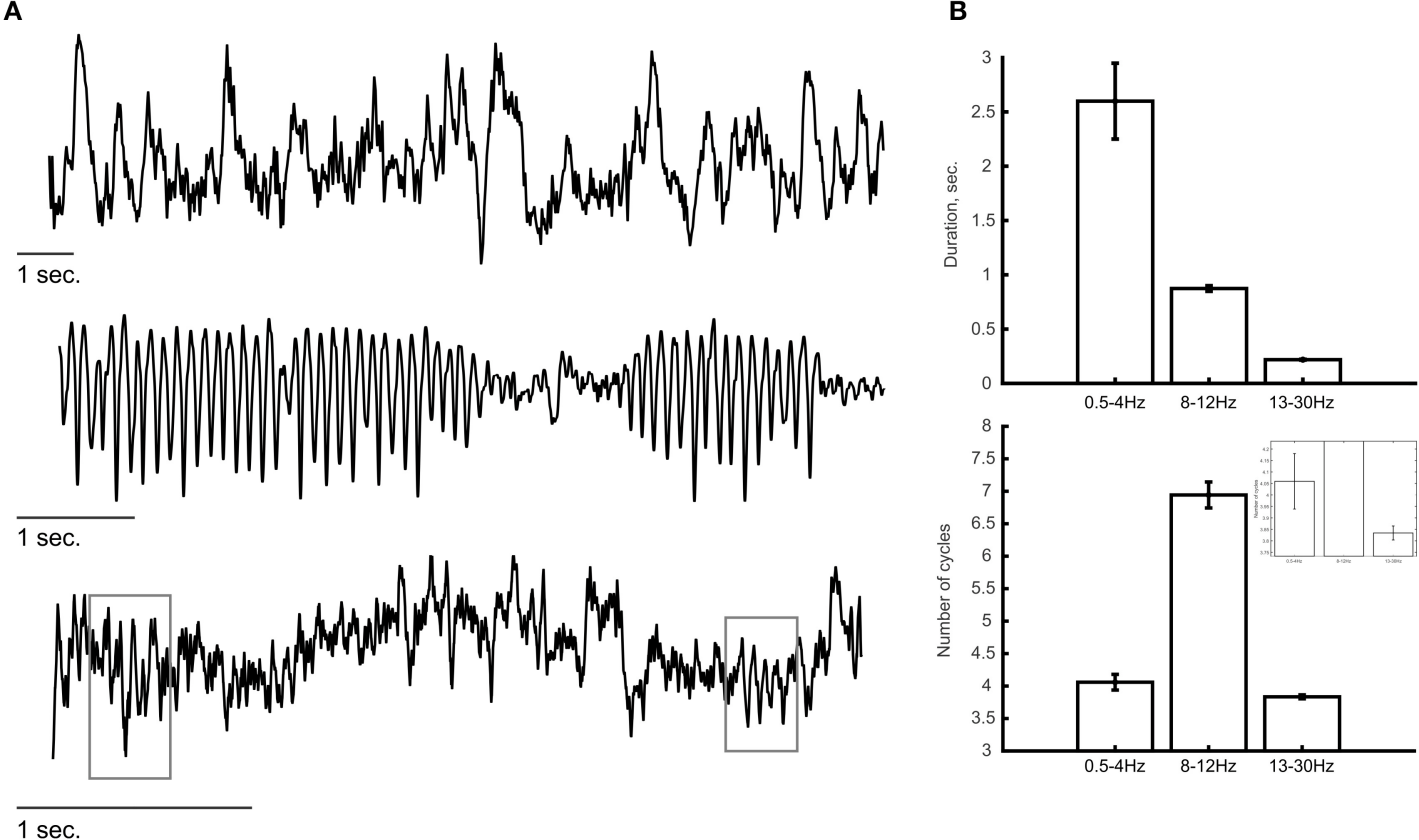
Examples of ongoing oscillatory activity. **(A)** Top and middle panels show a trace of invasive recordings from sEEG electrodes showing low-frequency oscillations (0.5–4 Hz, top; 8–12 Hz, middle). (Bottom) Laminar probe recording from a non-human primate showing bursts of oscillatory activity at a frequency of 13–30 Hz. Gray rectangles indicate the detection of transient oscillatory bursts. **(B)** Descriptive analysis of the duration of oscillatory activity at each frequency band. The duration of oscillations (top) was estimated as the period of time in which the power at that frequency band exceeded the 95th percentile of the theoretical χ^2^ distribution of wavelet power values. The number of cycles (bottom) were calculated by multiplying the duration (in seconds) by the peak frequency of the oscillation (in Hz). Lower frequency oscillations (i.e., 0.5–4 and 8–12 Hz) tend to show longer durations compared with the higher frequency oscillations (13–30 Hz) in which most oscillatory bursts consisted of ~3.5 cycles. The inset shows a zoomed version to visualize the differences between 0.5–4 and 13–30 Hz. Error bars indicate standard error of the mean.

Some of the methods mentioned above might reduce concerns regarding low SNR. Occasionally, we may observe oscillatory bursts of sufficient amplitude that the SNR is less of a concern (see e.g., below). There is no trivial way to define a duration value that can serve as a boundary between transient and sustained oscillations. Thus, we can only say that with most commonly used analysis approaches, particularly due to the practice of averaging multiple trials prior to quantification, oscillatory activity might appear longer than they actually are. Clearly, both the nature of the task performed by the subject and the recording technique (e.g., invasiveness, electrode location) would influence the amplitude, duration and frequency of the recorded oscillations and thus also the SNR. Thus, there might be substantial variability in the characteristics of these oscillations across different studies, when they are studied at the single-trial level. Typically, invasive recording techniques provide higher SNR than non-invasive techniques. Three independent examples of such invasive recordings in humans (sEEG) and non-human primates (laminar probe) are shown in Figure 2 where oscillations are visible by eye in ongoing recordings. Some of these oscillations tend to be more sustained, specifically at lower frequency (e.g., 8–12 Hz; Figure 2A, middle panel) while higher frequencies reveal a “bursty” profile in this example (13–30 Hz; Figure 2A, bottom panel). We estimated the duration and number of cycles at a descriptive level for a few minutes of recording from these example (Figure 2B; see figure caption for more details). Even though these are merely selected examples and should not be considered as evidence for the specific durations of oscillatory activity, such recordings give us confidence that the basic phenomenon of a bursty oscillation exists. Recently, Neymotin et al. (2020a) quantified oscillation event features in resting-state invasive recordings from auditory cortex of humans and non-human primates. To our knowledge, this was the first attempt to characterize ongoing (single trial) oscillatory activity across different frequencies and species. They found that oscillations at all commonly studied frequency bands (i.e., delta—high gamma) exhibit multiple cycles (average of four cycles across all frequency bands; range: 1–44 cycles) with fluctuating frequency and amplitude. They also found that ~90% of the time, oscillation events of at least one frequency band are occurring, suggesting that multicycle neuronal oscillations across a wide range of frequencies dominate auditory cortex dynamics. Interestingly, temporal predictability across bursts differed significantly from Poisson distribution assumption which indicates inter-burst quasi-rhythmicity.

However, none of the methods described above (including the ones that do not require a pre-defined threshold) can negate the possibility that a transient time-frequency profile results simply from low SNR (see Figure 1). Thus, the most convincing evidence suggesting that transient oscillatory activity exists and is meaningful come from studies relating features of single-trial oscillatory activity with behavioral or perceptual phenomenon and show that such signals add information on top of the traditional (e.g., averaged; sustained) view of neural oscillations.

### Oscillatory Activity as Transient Bursts

Classic evidence for sustained oscillatory activity were based on averaging neural signals across many trials to form a representation of the neural activity with higher SNR. However, moment to moment perceptual representations in the real-world do not operate in such a way. To understand the neural basis of perception and action, and the involvement of oscillatory activity in these processes, one must explore trial-by-trial changes in oscillatory dynamics. In the following section, we will review evidence for transient or “bursty” oscillatory activity in both resting state (ongoing) and task-related activity, as well as their relationship with behavior.

Transient EEG events were first described by Berger (1930) during sleep and were later termed sleep spindles (Loomis et al., 1935). These 12–14 Hz bursts of oscillatory activity have been found in all mammals and the thalamocortical mechanisms generating them have been well-established (Dijk et al., 1993; Steriade et al., 1993). Yet, their function remains unclear (see De Gennaro and Ferrara, 2003 for a review). The first observations of oscillatory activity as transient bursts in awake subjects dates to 1966 when Jaffe and Weiss reported unilateral alpha bursts that are different from the alpha rhythm in several aspects (Jaffe and Weiss, 1966). These “alpha-range bursts” appear mainly in temporal EEG electrodes and correlate with clinical evidence of brain disease. They last for 3–4 s and increase during hyperventilation or drowsiness. The authors report that activity of this type has been occasionally observed in their lab and listed as an unusual finding of unknown significance. Alpha bursts of ~3 s in duration were also found during periods of REM sleep (Cantero and Atienza, 2000). These bursts are different from sustained alpha in that they are not accompanied by an increase in EMG activity and thus might be indexing different functional role from REM background alpha. The authors hypothesize that such alpha bursts may work as a micro-arousal in human REM sleep to facilitate a connection between the dreaming brain and the external world. Transient oscillatory activity was also observed at lower frequency bands in humans. For example, Hebert and Lehmann (1977) found the emergence of theta bursts in healthy subjects practicing transcendental meditation. These bursts appeared every 2 min on average, had a duration of about 1.8 s and were preceded and followed by alpha rhythm. Since the subjects reported pleasant states during the theta bursts, the authors hypothesize that theta bursts may be the manifestation of a state adjustment mechanism that comes into play during prolonged low arousal states and related to relaxation. While these findings were reported for ongoing activity at different mental states, transient oscillatory activity at low frequencies was also observed in intracranial recordings in human epilepsy patients during a virtual reality environment navigation task (Bush et al., 2017). They reported that human theta oscillations appear in transient bursts that typically last several cycles around movement onset and throughout the movement, in contrast to the continuous rhythm in the rodent hippocampus (Watrous et al., 2013). Although it is not clear whether these sporadic oscillations could encode continuous self-motion information, it is possible that location estimates are updated intermittently during theta bursts, in accordance with the outcome of planned movements, rather than tracked continually throughout the movement by an ongoing theta oscillation.

In recent years, there has been a renewed interest in the bursty qualities of oscillatory activity, particularly in higher frequency bands. By analyzing the amplitude and frequency of gamma bursts above the auditory cortical regions of cats, Lakatos et al. (2004) found that while attention mostly affects amplitude, arousal affects the frequency of gamma oscillatory bursts. Sherman et al. (2016) found that spontaneous neocortical beta (15–29 Hz) from somatosensory and frontal cortex emerged as non-continuous beta events typically lasting <150 ms with a stereotypical waveform. These “beta events” occur with varying levels of alpha activity (that seemed more sustained) and their waveform seemed to be consistent across species (mice, monkeys, and humans). The authors determined that beta events do not necessarily depend on rhythmic inputs but on the relative timing and strength of synchronous proximal (i.e., proximal to the soma and basal dendrites) and distal (i.e., to apical dendrites in L2/3) drives. Beta bursts were also observed in local field potential (LFP) signals recorded from the striatum and motor–premotor cortex of macaque monkeys performing a reaching task (Feingold et al., 2015). Using single trial analysis, they showed that beta bursts typically lasted 90–115 ms, and that extended periods of beta band synchronization reflected a modulation in the density of these short bursts. Burst probabilities were region and task-time specific such that in motor cortex they peak following the movement, while in striatum they peaked after reward and continued through the post-performance period. Lundqvist et al. (2016) used a trial-by-trial analysis and found that brief bursts of gamma-band activity (45–100 Hz) accompanied encoding and re-activation of sensory information in recording sites associated with spiking that reflected “to be remembered” items. Neuronal activity reflecting encoding or decoding correlated with changes in gamma burst rate. Additionally, they showed that gamma—alpha (8–10 Hz) coupling was not related to the periodicity of the gamma bursts but rather to the consistency in the duration of the gamma bursts, indicating that lower frequencies might modulate gamma-burst duration. Beta band oscillations (20–35 Hz) also appeared as transients in the Lundqvist et al. findings and were interpreted as reflecting a default (holding) state because it was interrupted by encoding and decoding. The authors concluded that working memory is not associated with sustained activity but rather discrete oscillatory dynamics and spiking. Beta range oscillations were also suggested to serve to clear memory states by resonantly driving transient bouts of spike synchrony which destabilize the network activity (Schmidt et al., 2018). Interestingly, the most effective oscillatory activity for allowing flexible switching between network states was burst-like with a sharp onset rather than a pure sinusoid. In addition, the authors demonstrate that such oscillatory bursts arise spontaneously in networks of excitatory and inhibitory neurons.

Transient oscillatory events may also provide an additional coding space for neuronal processes by utilizing the rate or timing of the transient events with regard to the stimulus or even with regard to other transient events. Shin et al. (2017) showed that differences in the rate of beta events predicted detection of stimuli at perceptual threshold and that non-detectable trials were more likely to have a beta event within ~200 ms prior to the stimulus. Using MEG, Little et al. (2018) found that motor cortical beta in individual trials appears as high amplitude, transient infrequent bursts. Beta burst timing was a stronger predictor of single trial behavior than beta burst rate or single trial beta amplitude, with later bursts corresponding to delayed response times. The relative timing of transient events was studied in the context of sensorimotor synchronization using MEG to show that decoding of behavioral conditions using time-differences between transient events across brain regions is significantly more accurate than other characteristics of the signal (Tal and Abeles, 2016, 2018; Felsenstein et al., 2019). Moreover, such transient events (treated as a point-process) might form more complex repeating sequences of activation with millisecond precision (Tal and Abeles, 2016, 2018; Felsenstein et al., 2019). These results suggest that relevant information might be encoded by subtle time differences or cascades of transient events across the brain. Although the study of oscillatory activity as transient events is still at an early stage, several features of these bursts (e.g., rate, duration, timing) have been linked back to behavior suggesting that the dynamics of such transient activity may be involved in cognitive processes.

The current review focuses on oscillatory events, however, there are also transient events that are not oscillatory in nature. A clear example of such event is an evoked potential generated in response to a stimulus. The nature of harmonic analyses techniques, such as wavelets and Fourier transform identifies these signals as oscillations. The BOSC method (Caplan et al., 2001; Hughes et al., 2012) attempts to avoid the detection of transient, non-oscillatory events by identifying oscillatory episodes at each frequency using both power and duration thresholds. Another approach to avoid bias due to externally-driven events removed the average evoked-responses waveforms from each cortical layer (Neymotin et al., 2020a), though if this removal uses simple subtraction of the trial-averaged response from each single trial, it runs the risk of creating artifacts (Knuth et al., 2006). Tal and Abeles (2016) used an algorithm that may detect both oscillatory and non-oscillatory transient events using a template matching scheme. They show that most of their detected events were not associated with clear periodic oscillations. They demonstrate that both oscillatory and non-oscillatory events showed similar increases in population activity around the times of these events (Tal and Abeles, 2018) and suggested them as markers of sudden increase in population activity that might indicate the recruitment of a new cell assembly within the cortical patch. It is not yet clear whether event-related-potentials trigger the same mechanisms in terms of the canonical circuits activated by internally generated oscillations. Sherman et al. (2016) argued that such brief sharp events are due to brief, strong excitation of the superficial cortical layers riding on the broader but weaker excitation of deep cortical layers. Laminar biophysical models of the thalamocortical system that accurately simulate recorded signals, such as local field potentials, can be used to predict the types of waveforms that are recorded *in vivo* after sensory stimulation, and offer mechanistic explanations for their features. For example, providing brief, strong thalamocortical activation to a hypothetical neocortical model would trigger production of a transient ERP-like event in the circuit, with a characteristic waveform (Neymotin et al., 2020b). Although running a wavelet filter on such a waveform will produce high power at a frequency inverse to the duration of the ERP, since it was produced by a punctate event, this type of waveform should not be considered an oscillation (Neymotin et al., 2020a). In contrast, specific synaptic connectivity and input patterns provided to a circuit model lead to production of sustained multi-cycle oscillations. Some of these circuit mechanisms and their implications in detecting oscillatory bursts from electrophysiology data *in vivo* are described in the next section.

### Mechanisms of Transient Oscillatory Activity

**Figure 3:**
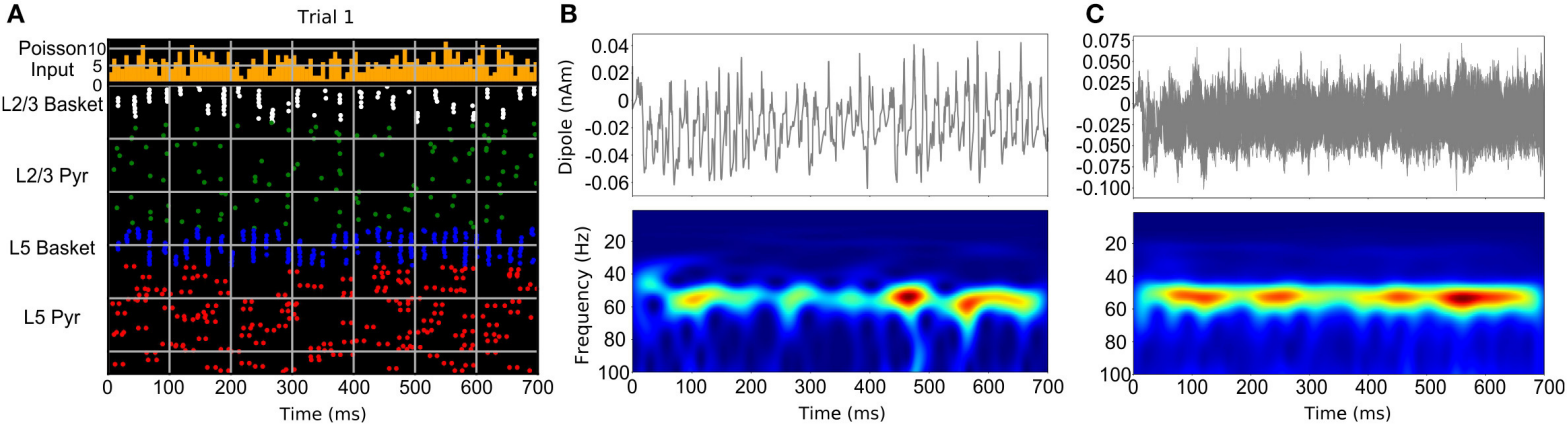
Neocortical circuit model used to simulate bursty gamma oscillation events through a weak PING mechanism. **(A)** Raster spiking plot of neuronal firing times from single trial of weak PING simulation. Top panel shows histogram of low-frequency, noisy Poisson inputs used to drive pyramidal neurons and interneurons in the model. Bottom panel shows population color-coded firing times of individual neurons. **(B)** Single trial current dipole signal (top) and Morlet wavelet spectrogram from dipole signal (bottom) from weak PING model. **(C)** Simulation of one hundred trials of weak PING model produces one hundred current dipole signals (top). Although gamma oscillation events occur at different times in each trial, averaging the wavelet spectrogram across trials (bottom) produces an appearance of a sustained gamma oscillation [adapted with permission from Figure 10 of Neymotin et al. (2020b) under the license: https://creativecommons.org/licenses/by/4.0/].

The studies discussed above suggest that neocortical oscillations tend to be short-lived and bursty, however, some neurological disorders, such as Parkinson's disease, are clearly associated with prolonged rhythms (Tinkhauser et al., 2017a,b). In general, stronger activation of a particular circuit component that generates a specific oscillation (such as gamma), would produce a more sustained form of that oscillation. Weaker activation, either through reduction of the frequency and strength of AMPA/NMDA synaptic inputs to that component or from stronger suppressive inhibition, can result in gaps between oscillatory bursts (Lee and Jones, 2013; Neymotin et al., 2020b). Figure 3 demonstrates the results of a neocortical column simulation of bursty Pyramidal-interneuron network gamma (PING) oscillations using the Human Neocortical Neurosolver (HNN) software (https://hnn.brown.edu). As shown in Figure 3, in PING, gamma is generated through a sequence of activations: (1) stochastic synaptic inputs drive spiking of pyramidal neurons, causing collateral activation of fast spiking (basket type) interneurons, (2) activation of the fast-spiking interneurons then causes feedback inhibition lasting a gamma cycle (~20 ms for 50 Hz gamma), determined by the duration of the rise and fall of the GABA_a_ synaptic conductance, and (3) after GABA_a_ inhibition runs its course, pyramidal neurons are again able to spike and the cycle repeats. Synchronization of inhibitory interneurons, which is responsible for generation of gamma rhythms, is seen in the raster plot of Figure 3A, with the nearly vertical lines that recur at a gamma period (white and blue). In general, sustained gamma is produced when there is continued strong activation of pyramidal neurons, which causes continuing periodic activation of the interneurons, resulting in large amplitude/persistent gamma. Lowering the frequency or strength of excitatory synaptic inputs driving the pyramidal neurons, causes weaker, intermittent activation of the interneurons, and temporal gaps between interneuron-generated gamma bursts, as shown in Figure 3A. Note that in the raster plot, not all interneurons are activated at each gamma bout. Additionally, different subsets of the interneurons are activated. This firing pattern is a hallmark of weak PING, considered weak because the gamma amplitude occasionally waxes and wanes depending on the level of interneuron activation. In this example, pyramidal neurons (green, red dots) fire even less frequently, but are still synchronized by the interneurons (note the periodic gaps between the sparse pyramidal neuron firing). Figure 3B shows a single trial of the current dipole signal generated by HNN's biophysical cortical circuit model (top) and its associated time-frequency representation, using the Morlet wavelet spectrogram (bottom). As shown, the current dipole signal's gamma oscillation has a peak between 40 and 60 Hz, and has power waxing and waning. A close look at the spectrogram reveals that the gamma oscillation is only present at discrete times. However, when taking the average wavelet spectrogram from multiple trials of this simulation (Figure 3C), the gamma oscillation appears to be more continuous. This is because the wavelet spectrogram always has positive values, and adding the gamma events which occur at different times across trials, produces the appearance of continuity. This is one mechanism for the bursty gamma observed in experimental data and highlights the importance of careful analysis of single trial data.

A related theme in cortical dynamics is the presence of multiple interacting oscillations caused by different time scales of inhibition provided by different classes of interneurons (for reviews see Whittington et al., 2000; Skinner, 2012; Kopell et al., 2014). For example, Neymotin et al. (2011) used a model with intermediate complexity to replicate normal and pathological hippocampal dynamics. In their model, oriens-lacunosum moleculare (OLM) interneurons produce theta through relatively long-lasting inhibition. OLM inhibition of fast-spiking basket and pyramidal neurons then modulated the faster gamma rhythm, which was produced through the standard PING mechanism. This interaction between OLM and basket interneurons caused gamma rhythms to increase and decrease based on the phase of the slower theta rhythm. This cross-frequency coupling mechanism could be used to model gamma bursts too, since a few strong cycles of gamma appear in between strong periods of OLM inhibition at the theta rhythm. As evidenced by a multitude of non-human (Lakatos et al., 2005; Buzsaki, 2006; Schroeder and Lakatos, 2009) and human (Canolty et al., 2006; Canolty and Knight, 2010) studies demonstrating phase-amplitude coupling, similar mechanisms should operate in the neocortex *in vivo*, which has an intricate circuitry with a multitude of interneuron types (Dienel and Lewis, 2019).

Another circuit model of neocortex aimed at determining the origin of beta oscillation events (Sherman et al., 2016). This biophysical model simulated current dipole signals produced by the circuit, allowing explicit comparison to source-localized current dipole signals from MEG/EEG studies. The neocortical model consisted of simplified models of pyramidal and inhibitory interneurons arranged in superficial and deep cortical layers and interconnected using AMPA and GABA synapses. Pyramidal neuron dendrites spanned the cortical layers. Synaptic inputs were provided to the pyramidal neurons to initiate network activity. These synaptic inputs were applied at proximal and distal locations on pyramidal neuron dendrites to model inputs from thalamic core (proximal) and thalamic matrix and corticocortical feedback (distal). Each of these types of synaptic input pushed current flow within the pyramidal neuron apical dendrites in opposite directions. The model was able to produce beta oscillation events through a series of ~10 Hz stochastic synaptic inputs provided to proximal and distal pyramidal neuron dendrites. Beta oscillation events were produced when ~100 ms duration proximal synaptic inputs (pushed current flow toward superficial layers) were truncated by a more synchronous 50 ms distal inputs (sharply pushed current flow toward deep layers), which produced a 50 ms current dipole waveform, matching the waveforms seen from source-localized human MEG experiments. Since the synaptic inputs were stochastic, the production of beta events was also stochastic, producing bursty oscillation events. Additionally, when the proximal and distal synaptic inputs arrived out of phase, instead of beta, alpha events were produced. This occurred because each set of synaptic inputs was provided to the model at the alpha period (100 ms interval). Invasive laminar electrophysiology recordings from non-human primate somatosensory cortex were used to confirm the model's accuracy.

## Concluding Remarks

We reviewed several studies suggesting that brain rhythms tend to appear as short-lived bursts of oscillatory activity. The importance of these observations lies in our interpretation of the functional role of neural oscillations, the mechanisms generating them, the potential information they may carry, and the way we must analyze them. One of the major points raised here is that sustained oscillations can appear “bursty” in the presence of noise and conversely, “bursty” oscillations can appear sustained when averaged across trials. Many cognitive studies report an increase in oscillatory activity that only lasts for a brief duration (e.g., a few tens of milliseconds). This might represent the main response to the stimulus but at the same time, might fail to explain the trial by trial variance in burst timing, frequency, and amplitude. In addition, the averaging approach might also miss other important responses that are either “smeared” in the averaged response or appear at different times across trials and are obscured by averaging. While the averaging approach has its advantages, specifically in increasing the SNR of the phase-locked response to a stimulus, single-trial analysis of oscillatory events should be performed to better understand the mechanisms of oscillatory activity by exploring the variability in different features of oscillatory activity across trials and, whenever possible, their relationship with behavioral and perceptual phenomenon. Estimating the characteristics of single-trial (or ongoing) oscillations is not trivial, and thus several methods for detecting transient events were suggested. Most of these methods depend on amplitude and duration thresholds or a probabilistic threshold. Due to the differences in the goals of each study, the design of the experiments, the recording techniques, and the frequency and time-windows studied, variability in the estimation of the characteristics of single-trial oscillations is to be expected and it is difficult to provide a single recommendation on the algorithm that should be used to study neuronal rhythms at the single trial. The simplest approaches (such as p-episodes) can be useful in cases where lower computational time is essential (such as in closed-loop experiments), while more computationally demanding approaches can achieve more fine-tuned results offline. When possible, we recommend applying more sophisticated algorithms, such as HMM or amplitude and duration thresholds combined with template-matching that carry less risk of false detection due to artifacts or noise. We identify five features of short-lived oscillations that may provide information-coding space for the brain: (1) Amplitude—indicates the size and synchronicity of the underlying neuronal population. (2) Temporal span (duration)—how long the synchrony within a population is maintained. (3) Frequency span—might index the participant neuron circuits and critically, the inherent conductances of their specific neuronal constituents. When studied at the single-trial level, these features may explain variability in behavioral performance across trials that cannot be observed in the averaged waveforms. (4) Inter-burst and stimulus-burst interaction–measures, such as the burst-rate, burst-timing, inter-burst interval, coefficient of variation, fano-factor and more complex spatio-temporal sequences comprised by transient bursts might be used to explore the single trial dynamics of oscillatory activity and non-oscillatory transient events. The general idea is to treat the timing of these transient events as a parallel point-process to study their temporal relationship with other events and with the stimulus. For example, determine the rhythmicity across events from a given oscillation frequency band (e.g., whether oscillatory events are rhythmic and predictable, or Poisson distributed). (5) Time-domain waveforms—might index different biophysical generators. We note that this feature is more abstract and challenging to measure but should be further studied to extract the meaningful features within the waveforms and test for repeating waveforms in the data. Time-domain waveforms may also reveal differences between oscillatory and non-oscillatory events that might differ in both their mechanisms and their role in information processing. Such features are necessary to study brain rhythms at the single trial level and take advantage of the temporal dynamics of neural oscillations to better understand their role in information transmission, processing, and coding.

## Author Contributions

IT and SN performed the simulations. SN designed the model shown in Figure 3. SB and CS provided the data for the examples shown in Figure 2. IT and CS wrote the manuscript with support from SN, SB, and PL. All authors contributed to the article and approved the submitted version.

## Conflict of Interest

The authors declare that the research was conducted in the absence of any commercial or financial relationships that could be construed as a potential conflict of interest.
